# Multiplex imaging reveals the architecture of the tumor immune microenvironment

**DOI:** 10.20892/j.issn.2095-3941.2021.0494

**Published:** 2021-10-29

**Authors:** Junlei Zhang, Jinyuan Song, Jianpeng Sheng, Xueli Bai, Tingbo Liang

**Affiliations:** 1 Department of Hepatobiliary and Pancreatic Surgery; Zhejiang Provincial Key Laboratory of Pancreatic Disease, the First Affiliated Hospital, Zhejiang University School of Medicine; Zhejiang University Cancer Centre, Zhejiang University, Hangzhou 310002, China

The tumor immune microenvironment (TME) is composed of a variety of components, such as tumor cells, immune cells, and the extracellular matrix. The TME has been studied through transcriptomic, proteomic, metabolomic, and phosphoproteomic approaches, which have provided researchers with a wealth of TME-related molecular information.

Detecting immune cell markers and their immune status is the focus of research on changes in immune cells in the TME. For most immune cell analysis methods, such as traditional fluorescence flow cytometry and the relatively novel mass spectrometry flow cytometry, spatial information on cells is lost during sample preparation. Immunohistochemistry (IHC) and immunofluorescence (IF) imaging can provide simultaneous positional signals for only limited numbers of components.

Exploring the positional relationships among more components of the microenvironment and their roles in tumorigenesis, cancer development, and drug responses is particularly important. Conventional IHC or IF, because of their limited multiplexing capacity, cannot satisfy these demands. Therefore, several recent methods to achieve multiplex imaging have been developed. For instance, imaging mass cytometry (IMC) overcomes the spectral overlap of fluorophores by virtue of their metallic character^1^. Alternatively, DNA-barcoding methods have been explored to achieve multiplexing. Co-detection by indexing (CODEX) applies dozens of antibodies labeled with orthogonal DNA barcodes followed by polymerization of fluorescent probes *in situ*^2^. On the basis of DNA-barcoding, immunostaining with signal amplification by exchange reaction (Immuno-SABER) offers independent programmable signal amplification, which enhances the sensitivity of low-abundance targets^3^.

With the development of new methods, related analysis software has also been established to enable extraction, quantification, and presentation of highly multiplexed information. In several tumor fields, multiplex imaging has played a crucial role in understanding the TME and improving anti-tumor strategies.

The recently developed methods can be divided into 2 major types according to whether the reporter is fluorescent. IMC, multiplexed ion beam imaging (MIBI)^4^, and multiplexed vibrational imaging^5^ are the representative non-fluorescent techniques. In contrast, CODEX, Vectra Polaris and Immuno-SABER use fluorescent reporters to detect various markers.

IMC extends the multiplex analysis capabilities of cytometry by time of flight (CyTOF)^6^ to measure spatial cellular information. A unique rare earth metal is conjugated to an antibody to replace fluorescent markers, and dozens of metal-labeled antibodies are simultaneously incubated with tissue sections. After a series of washes, the tissue slides are positioned in a laser ablation chamber and ablated spot by spot^1^. A mixed argon and helium stream transports the ablated material to the mass cytometer (**Figure 1A**). Mass cytometry uses a time-of-flight inductively coupled plasma mass spectrometry (ICP-MS) instrument that can detect various metals simultaneously^6^. IMC can exploit more than 40 metal-labeled antibodies incubated on one slide at the same time. Theoretically, more than 100 markers can be detected simultaneously with IMC^6^. The speed of image acquisition is 100 min per 1 square millimeter at 1 µm resolution^1^. Therefore, scanning a 1 square-centimeter tissue section requires nearly 7 days, thus limiting large-scale imaging.

**Figure 1:**
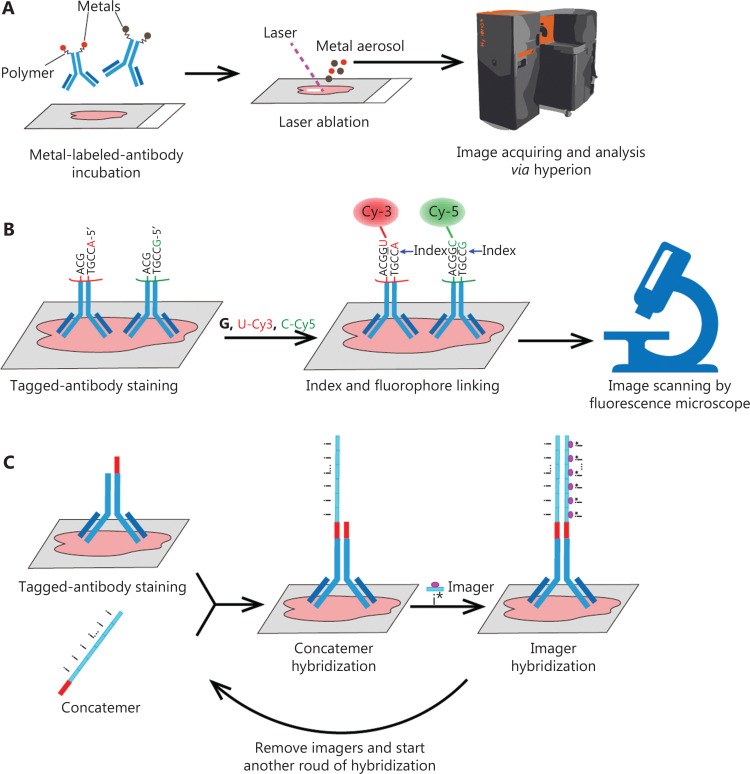
The workflows of 3 types of multiplex imaging methods. (A) Imaging mass cytometry (IMC) acquires tissue spatial information *via* laser ablation of cells combined with metal-labeled antibodies. (B) Co-detection by indexing (CODEX) scans images through rounds of fluorophore and index staining, and removal. (C) Immunostaining with signal amplification by exchange reaction (Immuno-SABER) provides highly multiplexed amplification capability that can be tuned independently for multiple targets by controlling the length of the concatemers and the number of imagers.

The CODEX technique uses a different mechanism based on conventional fluorescence imaging. In contrast to classical methods, fluorophores do not form covalent bond with antibodies to directly label the targets. All purified antibodies are tagged with a uniquely designed oligonucleotide, and, as in IMC, all labeled antibody mixtures are incubated with tissues. The cells are then exposed to a nucleotide solution including 1 of 2 non-fluorescent “index” nucleotides and 2 fluorescently labeled nucleotides. The first position across all tagged antibodies bonded to cells is filled with the “index” nucleotides. The DNA tags linked to fluorophores and the barcodes on the antibodies are in one-to-one correspondence, and the fluorophores and antibodies successfully dock with each other only if the index nucleotide previously incorporated fluorescent dNTPs. Those 2 antibodies are then imaged through fluorescence microscopy. After 1 cycle, the fluorophores are cleaved and washed away, and another index nucleotide is used in the next cycle. At the end of the experiment, the multiparameter image can be reconstructed^2^. Fluorescence microscopy, compared with mass cytometry, dramatically accelerates the imaging procedure (**Figure 1B**).

Immuno-SABER increases the detection sensitivity by multiplexed signal amplification, and each signal can be tuned independently for multiple targets. Immuno-SABER achieves signal amplification through orthogonal DNA concatemers produced *via* a primer exchange reaction^3^. The amplification is achieved through the hybridization of concatemers with tagged antibodies, followed by the incubation of imagers, thereby facilitating the imaging of low-expression targets (**Figure 1C**).

Another technique, Vectra Polaris (Akoya Biosciences, Marlborough, MA, USA), can detect fewer than 10 markers simultaneously. In this method, a tyramide signal amplification (TSA) system amplifies the signal through covalent binding of diffusive tyramide molecules in the vicinity of the target^7^. The stained sections are scanned with the Vectra System (PerkinElmer, Waltham, MA, USA), which captures the fluorescence spectra at 20-nm wavelength intervals from 420 to 720 nm with identical exposure times. Then all single-color images are combined to construct a multiplexed image^8,9^. However, TSA amplifies only one target per round, and long times are required for multiple rounds of microwave-based antibody removal to achieve spectral multiplexing.

The greatest advantage of these novel methods over conventional imaging methods is that as many as 50 biomarkers can be detected simultaneously on one slide. Therefore, dozens of cell subtypes can be identified *in situ*. According to the spatial information of cell subtypes, the interactions among these cells can be analyzed. However, conventional imaging methods can detect only several markers and consequently identify the relationships among 2 or 3 cell types. In addition, the TME is very complex, comprising many cell subtypes and the extracellular matrix. Using only several markers cannot meet research needs, and thus high-throughput imaging technologies are necessary to analyze the tumor architecture. Multiplex imaging has another advantage in that it requires less tumor tissue than conventional analyses; this capability is important for research on rare tumors, because of their scarcity. One pathological section is sufficient to reveal the microenvironment of one tumor tissue. In addition, imaging signals are improved through these new methods. Metals can amplify the signals of the first antibodies. Immuno-SABER can even control every marker’s signal strength separately. Because of these advantages, novel multiplex imaging is a promising technique for research on tumor immunology.

With the rapid development of new technologies, such as single-cell RNA sequencing (scRNA-seq) and CyTOF mass cytometry^6,10,11^, researchers have been able to gain a comprehensive understanding of the heterogeneity of various tumors. Many subtypes of immune cells that play important roles in the TME have been identified. Nevertheless, tissues are dissociated in these 2 methods, and neither method can reveal the spatial information and neighboring cell interactions. Therefore, multiplex imaging techniques enable researchers to determine tumor spatial information and explore the TME in depth. In addition, a variety of related analysis software has been developed to help researchers analyze results more efficiently and accurately.

A series of studies that discovered new targets to improve tumor-treatment strategies are summarized in **Table 1**. Hepatocellular carcinoma (HCC), the most common primary malignancy of the liver, threatens the lives of many people^16^. Liu et al.^8^ have used TSA systems to reveal that the expression of programmed cell death 1 (PD-1) correlates with patient prognosis in HCC. In addition, Zheng et al.^15^ have used the same method to reveal the landscape of infiltrating T cells in liver cancer. Recently, using IMC, Sheng et al.^17^ have highlighted the potential to target Kupffer cells as a novel immunotherapeutic approach to treat HCC. A 36-biomarker multiplexed IMC panel was designed to scan tissues from hundreds of patients. The histological structure of HCC was portrayed perfectly by pan-cytokeratin, CD31, alpha smooth muscle actin (α-SMA), and collagen I. Compared with hematoxylin and eosin staining, IMC marked hepatic sinusoids, hepatic cords, and portal areas more distinctly and recognizably. MCD viewer was developed by Fluidigm (San Francisco, CA, USA) to present multi-color images. Then CellProfiler software (the Whitehead Institute for Biomedical Research and MIT’s MIT Computer Science and Artificial Intelligence Laboratory) was used to segment cells, and histoCAT^12^ software was used for t-distributed stochastic neighbor embedding (tSNE) and PhenoGraph analyses. Schapiro et al.^12^ developed an open-source computational histological imaging cytometry analysis toolbox, histoCAT, to enable interactive, quantitative, and comprehensive exploration of cell phenotypes, cell-cell interactions, immune microenvironments, and morphological structures within intact tumors. They discovered the influence of Kupffer cells in suppressing T cells and driving surrounding PD-1 overexpression. Sheng et al.^17^ have also introduced the concept of cellular neighborhoods (CNs), functional units organized from diverse cellular components. The authors have defined the CN of HCC while preserving the TME architecture and, on that basis, have identified potential targets for novel immunotherapy. Previously, how Kupffer cells or other cell types influence the surrounding components of a tumor was unknown, and representing their interactions was challenging. With multiplex imaging and related analysis tools, these problems can be resolved.

**Table 1:** Summary of multiplex imaging methods, related analytical software, research fields, and new targets discovered *via* these technologies

Methods	Analytical software	Research fields	New therapeutic targets
^†^IMC (Hyperion)^1^	CellProfiler, histoCAT, Voronoi diagrams	HCC	Resident macrophages^12^
		Breast carcinoma	Vimentin^+^Slug^−^ macrophages^13^
		Melanoma	B2M^14^
^‡^CODEX^2^	Volumetric segmentation algorithm by Yury Goltsev et al.^2^, X-shift	Colorectal cancer	PD-1^+^CD4^+^ T cells^15^
^§^Immuno-SABER^3^	–	Imaging of normal human tissue	–
^¶^TSA systems (Vectra Polaris)^7^	Nuance and InForm image analysis software (PerkinElmer)	HCC	Treg and exhausted CD8 T cells^16^, PD-1^8^

^†^IMC, imaging mass cytometry; ^‡^CODEX, co-detection by indexing; ^§^Immuno-SABER, immunostaining with signal amplification by exchange reaction; ^¶^TSA systems, tyramide signal amplification system.

Notably, the concept of the CN was also established by Christian et al.^18^, as represented by Voronoi diagrams^19^. The authors explored the TME at the colorectal cancer (CRC) invasive front *via* CODEX. A 56-biomarker panel was established to depict various CNs, which revealed the spatial organization of the CRC TME. Using this novel tool, the authors divided patients into low- and high-risk groups. The latter showed local enrichment of PD-1^+^CD4^+^ T cells, which correlated with patient survival and provided another specific target for CRC therapy^18^.

Breast carcinoma is the leading cause of cancer-related death in women^20^. Multiplex imaging has also become a powerful tool to gain insights into the TME of breast cancer and potentially enable more precise treatment. Ali et al.^13^ have used IMC to quantify the expression of 37 proteins at subcellular spatial resolution in more than 400 tumors from the METABRIC cohort. They have customized an image processing pipeline (https://github.com/BodenmillerGroup/imctools) and used CellProfiler to quantify single-cell signals and identify CNs. The authors have revealed both the composition and architecture of breast tumor ecosystems. Jackson et al.^21^ have also used IMC to analyze the pathological landscape of breast carcinoma. They have concluded that pathology in single-cells performs better in segregating patients with distinct clinical outcomes than the current strategy of clinical classification.

Moreover, the study of other types of cancers, such as melanoma^22,14^ and pancreatic duct cancer^23^, has benefited from these novel technologies. The discoveries made through multiplex imaging have provided a set of new targets that may be used to develop novel treatments for cancers.

## Perspectives

This review summarizes the most recent multicolor imaging technologies enabling visualization of dozens of biomarkers. These novel methods pave the way to insights into the heterogeneity of various tumors, particularly the immune microenvironment. Spatial cell-type distribution, cell-cell interactions, and CNs can be revealed by multiplex imaging. Many researchers have used these different methods to conduct studies on tumors, such as HCC, breast carcinoma, and melanoma. Researchers can use these advanced techniques to reveal different patterns of immune reactions, such as how tumors recruit immune cells or how immune cells change their states in the TME. These methods are also valuable in clinical pathology. With abundant pathological information, tumors can be staged more precisely to guide treatment strategies and improve prognosis. However, some challenges remain to be overcome. Low scanning speed, unsatisfactory resolution, and high cost restrict these the wider application of these methods in clinical screening, diagnosis, treatment, and prognostication. Furthermore, 2-dimensional imaging is insufficient to reveal tumor heterogeneity. Future research might focus on the 3-dimensional imaging of tumors, which would enable more precise and comprehensive exploration of the inner tumor microenvironment. We hope that multiplex imaging can be developed into more powerful tools for scientific researchers and clinicians in the fight against tumors.
